# Halotolerant *Exiguobacterium profundum* PHM11 Tolerate Salinity by Accumulating L-Proline and Fine-Tuning Gene Expression Profiles of Related Metabolic Pathways

**DOI:** 10.3389/fmicb.2018.00423

**Published:** 2018-03-12

**Authors:** Vikas K. Patel, Ruchi Srivastava, Anjney Sharma, Anchal K. Srivastava, Savita Singh, Alok K. Srivastava, Prem L. Kashyap, Hillol Chakdar, K. Pandiyan, Alok Kalra, Anil K. Saxena

**Affiliations:** ^1^Laboratory of Genomics, ICAR-National Bureau of Agriculturally Important Microorganisms, Maunath Bhanjan, India; ^2^Division of Plant Pathology, ICAR-Indian Institute of Wheat and Barley Research, Karnal, India; ^3^Department of Microbial Technology, CSIR-Central Institute of Medicinal and Aromatic Plants, Lucknow, India

**Keywords:** *Exiguobacterium profundum* PHM11, salinity, gene expression, metabolic pathways, beta-carotene

## Abstract

Salinity stress is one of the serious factors, limiting production of major agricultural crops; especially, in sodic soils. A number of approaches are being applied to mitigate the salt-induced adverse effects in agricultural crops through implying different halotolerant microbes. In this aspect, a halotolerant, *Exiguobacterium profundum* PHM11 was evaluated under eight different salinity regimes; 100, 250, 500, 1000, 1500, 2000, 2500, and 3000 mM to know its inherent salt tolerance limits and salt-induced consequences affecting its natural metabolism. Based on the stoichiometric growth kinetics; 100 and 1500 mM concentrations were selected as optimal and minimal performance limits for PHM11. To know, how salt stress affects the expression profiles of regulatory genes of its key metabolic pathways, and total production of important metabolites; biomass, carotenoids, beta-carotene production, IAA and proline contents, and expression profiles of key genes affecting the protein folding, structural adaptations, transportation across the cell membrane, stress tolerance, carotenoids, IAA and mannitol production in PHM11 were studied under 100 and 1500 mM salinity. *E. profundum* PHM11 showed maximum and minimum growth, biomass and metabolite production at 100 and 1500 mM salinity respectively. Salt-induced fine-tuning of expression profiles of key genes of stress pathways was determined in halotolerant bacterium PHM11.

## Introduction

*Exiguobacterium*, a well known halotolerant bacteria, has been attracting most of the researchers as being a vital agent of salinity stress mitigation and an important source of useful carotenoids (Rodrigues et al., 2008). Different species of *Exiguobacterium* such as *E. alakliphilum, E. antarkticum, E. indicum, E. mexicanum, E. oxidotolerans, E. sibiricum, and E. undae* have been identified from the diverse habitats; however, a few one have significant halotolerant features (Bharti et al., 2013). Salinity stress is major issue affecting the production of a number of agricultural crops (Qados and Amira, 2011; Jaarsma et al., 2013). Some crop varieties are highly prone to salinity that alternatively affects their production in sodic soils. Efforts are continuously ongoing at global scale to improve the production of different salt sensitive crops in sodic soils through implementing the modern approaches of biotechnology including; development of novel plant varieties by plant breeding and synthetic biology, regular searches for salt mitigating endophytes and plant growth promoting bacteria and engaging them in plant growth promotion under salinity stress (Bharti et al., 2016), microbial synthetic ecology to get the high-salt mitigating and plant growth promoting bacterial consortia (Ryu et al., 2003; Malusa et al., 2012; Farrar et al., 2014), and use of nanotechnology (Saxena et al., 2016). Consequently, a great success has been achieved in getting more biomass under salinity through these approaches (Bharti et al., 2016; Abdel Latef et al., 2017). Yet, implementation of halotolerant bacteria for promoting the plant growth in sodic soil has given significantly better results. A number of transcriptomic studies of plants inoculated with endophytes and plant growth promoting rhizobacteria have been done and still ongoing; however, there is a long research gap in knowing that how these useful halotolerant bacteria are fine-tuning their gene expression profiles to acclimatize, and what physiological changes are happening that alternatively promoting their survival under salinity. A quick literature search opens a lot of published researches about the identification of halotolerant bacteria, their characterization and role in salinity stress mitigation on plants. However, till date, very little efforts have been made to know the real consequences affecting the inherent physiology of halotolerant bacteria through modulating the expression profiles of key genes of their important regulatory pathways. To explore this, a halotolerant *E. profundum* PHM11 bacterium was isolated and characterized for different plant growth promotion traits and tested for the changes in its physiology and gene expression patterns under salinity. This study reflected the hidden changes in the modulation of gene expression patterns of this novel bacterium under salinity. Previously, PHM11 was tested for its plant growth promotion traits on a number of agricultural crops and given significant results. In this study, efforts were made to get the salt-induced changes in the physiology and gene expression profiles of PHM11 under salinity that alternatively affect its survival, stress mitigation efficiency, and plant growth promotion potential.

Considering the importance of transcriptomics in gathering the hidden changes in depicting the salinity tolerance by halotolerant bacteria, we performed the comparative physiological and gene expression studies of *E. profundum* PHM11 under two different regimes of salinity with non-saline controls, and tried to get the molecular and physiological consequences affecting the salt-tolerance of PHM11. Key genes of stress mitigating pathways of PHM11 such as production proline, mannitol and other stress protecting compounds, plant growth promoting bio-molecules, and secretion related proteins were evaluated to find out the real consequences that are happening in salt-affected PHM11 cells.

## Materials and Methods

### 16S rRNA Gene Sequencing and Identification of Bacterium PHM11

Halotolerant *E. profundum* PHM11 was originally isolated through routine bacterial isolation techniques from the sodic soil of Babatpur, Varanasi, UP, India. The organism was isolated and maintained on the nutrient agar media supplemented with 5.0% sodium chloride. Auxenic culture was preserved in 40% glycerol at -80°C. The 16S rRNA gene was amplified according to the Awasthi et al. (2011) by using the universal bacterial primers 16S PF 5′-AGAGTTGATCCTGGCTCAG-3′ and 16S PR 5′-AAGGAGGTGATCCAGCCGCA-3′ and sequenced by using Big Dye^®^ Terminator v3.1 cycle sequencing kit (Applied Biosystems, USA) on a 3130xl Genetic Analyzer (Applied Biosystems, United States). Nucleotide Blast (BLASTn) analysis was performed for the obtained 16S rRNA sequence of PHM11 (Altschul et al., 1990). Later, complete 16S rRNA gene sequence was retrieved from the originally sequenced whole genome of PHM11 to further confirm the accurate species level identification (whole genome accession: NZ_MRSV01000001).

### Stoichiometric Growth Kinetics

The relationship between corresponding optical density at 600 nm wavelength (OD6_00_) and *E. profundum* PHM11 cell density was determined by generating a sigmoidal, logistic regression model. Hemocytometer was used for total count of the cells while viable cell count was performed through cell counting by determining the colony forming units. The non-linear sigmoidal logistic regression equation was used to establish the relationship between optical density (OD_600_) and cell counts.

$$y=y_{0}+\frac{a}{1+{\left(\frac{x}{x_{0}}\right)}^{b}}$$

where; ‘*y*’ is the PHM 11 cell density at a given O.D_600_, ‘*y*_0_’ is the initial cell density and ‘*x*_0_’ and ‘*x*’ are initial and final OD values at the 600 nm. Subsequently, in the regression equation of the line; an absolute value of the slope is the parameter “*b*”, and the intercept of the line is the parameter “*a*”.

### Evaluation of Salinity Tolerance Limits

A single colony of *E. profundum* PHM11 was inoculated in 50 mL of nutrient broth (NB) solution, grown overnight at 28°C and 150 rpm. Exponentially grown PHM11culture (1 × 10^8^ cells) was inoculated in 100 mL sterile NB containing different concentrations of sodium chloride (NaCl); 100, 250, 500, 1000, 1500, 2000, 2500, and 3000 mM in 250 mL in conical (Erlenmeyer) flasks, and grown at 28°C, 150 rpm for 72 h. Absorbance of different salt-treated and non-saline control cultures was measured at the interval of each 1 h at 600 nm. Measurements were done until harvesting of cultures. At last, values of cell density and optical density were extrapolated to get the actual cell density of different flasks. Based on the growth rates and observations; number of generations, mean generation time, specific growth rates and maximum specific growth rates were calculated (Prescott et al., 2002; Patel et al., 2016). Considering the growth capacity of PHM11 under different salinity levels; 100 and 1500 mM salt concentrations were selected as minimal and optimal regimes, and compared with control PHM11 for its growth and biomass production, and other physiological features.

### Bacterial Physiology

To get the real impact of selected regimes of salinity on PHM11, biomass yields, carotenoids and beta-carotene production, IAA and total proline of 100 and 1500 mM grown cells were compared with the experimental control PHM11 cells. Biomass was quantified gravimetrically (Patel et al., 2014), carotenoids were extracted according to Jensen (1978), beta-carotene was purified through separating the total carotenoids on thin layer chromatography (TLC), and later on quantified by measuring the absorbance at 450 nm, and extrapolating the obtained OD values with its original standard (Miller et al., 1935). The standard curve of beta-carotene was prepared by dissolving 0.1 mg mL^-1^ beta-carotene in methanol (Zscheile et al., 1942). Concentrations of phytohormone auxin (IAA) was determined from equal volumes while stress marker L-proline was quantified from equal biomass of 100 and 1500 mM salt-treated and control *E. profundum* PHM11 cultures by following the standardized methods suggested by Gordon and Weber (1951), and Bates et al. (1973) respectively.

### Total RNA Isolation

Total RNA was isolated from *E. profundum* PHM11 through guanidium thiocyanate (GTC) method (Chomczynski and Sacchi, 2006) with slight modifications. All the materials used for RNA isolation were treated with diethylpyrocarbonate (DEPC), and all the reagents were prepared in sterilized DEPC water. Approximately 50 mg cells of different salt-treated and non-saline control PHM11 cells were taken and washed twice in TE buffer in 2 mL microcentrifuge tubes (MCTs) and added 500 μL denaturing solution containing; 4 M GTC, 0.5% sarkosyl, 25 mM sodium citrate (pH 7.0, adjusted with HCl), followed by addition of 10 μL of lysozyme from 0.1 mg mL^-1^ stock solution and incubated at room temperature (RT) for 30 min, followed by the addition of 100 μg of DNaseI to remove the DNA contamination. After that 3.6 μL of β mercaptoethanol, and 50 μL of 2.0 M CH_3_COONa (pH 4.0) and 500 μL of water-saturated phenol were added and mixed gently. 100 μL of chloroform: isoamyl alcohol (49:1) was added and mixed through inverting the tubes which were later incubated for 15 min at 4°C in ice. The aqueous phase was separated by centrifugation at 10,000 rpm for 20 min at 4°C and collected in fresh sterile MCT, added 500 μL isopropanol, and incubated at -20°C for one h to precipitate RNA. Each of RNA pellet were washed with 75% ethanol by centrifugation at 10,000 rpm for 10 min at 4°C and pellets were dried for 40 min and finally dissolved in 50–100 μL triple autoclaved DEPC MQ water at 4–15°C for overnight. The quality of RNA was checked by 1.0% formaldehyde agarose gel electrophoresis.

### cDNA Synthesis

cDNA was synthesized from 3.0 μg of RNA of non-saline control, 100 and 1500 mM salt-treated PHM11 cells using a first strand cDNA synthesis system (Thermo Scientific) and random hexamer primers.

### Primer Designing

From originally sequenced and annotated whole genome of *E. profundum* PHM11, complete open reading frames (ORFs) for different genes were retrieved for primer designing. Based on the published literature database of *Exiguobacterium* and related organisms, various genes were selected from *E. profundum* PHM11 genome by considering their roles in salinity mitigation, structural adaptations and important metabolites production under stress. For deciphering the modulation of gene expression profiles of key enzymes under different regimes of salinity, quantitative real-time PCR (qPCR) primers were designed using Primer express 7.0 tool to get the amplicon sizes in the range between 100 to 150 bp for different genes (Singh and Pandey, 2015), and validated *in silico* through nucleotide blast (BLASTn) and primer-blast analysis respectively for their accuracy and amplicon sizes (Altschul et al., 1990).

### Gene Expression Studies

To get the real picture of gene expression profiles of key genes encoding for the enzymes of IAA biosynthesis pathway, proline biosynthesis pathway, mannitol biosynthesis pathway, secretion pathway, carotenoid/lycopene biosynthesis pathway, shape determination, protein folding related chaperons, transcription and translation were elucidated from the salt-treated and control cDNA samples of *E. profundum* PHM11 through real-time quantitative approaches. Each cDNA sample was diluted ten times using nuclease free MQ water, and 1.0 μL of diluted sample was considered for performing the qPCR experiments. List of different primers used for the relative quantification of various biosynthetic gene transcripts are given in Supplementary Table 1. Name of genes and their corresponding roles are given in **Table 1**. Each 20-μl PCR reaction mixture contained SYBR Green I (stock diluted 1:20,000) and 0.5 U of AmpliTaq DNA polymerase (Applied Biosystems, Foster City, CA, United States). The final concentrations of Mg^2++^ and primers were 4 mM and 0.5 μM, respectively.

**Table 1:** Genes selected from the whole genome of *E. profundum* PHM11 to study their expression profiles under salinity.

**Transcription and translation**	
*nusG*	Transcription antitermination protein nusG
*rsbW*	SigmaB stress response regulation;isu, Serine-protein kinase RsbW
*marR*	Transcriptional regulator MarR
*sigR*	RNA polymerase sigma factor SigV
*sphR*	Phosphate regulon transcriptional regulatory protein PhoB (SphR)
**Protein folding**	
^-^*dnaK*	Chaperon protein DnaK
*dnaJ*	Chaperone protein DnaJ
*hspGroES*	Heat shock protein 60 family chaperon GroES
*hspGroEL*	Heat shock protein 60 family chaperon GroEL
**Structural adaptations**	
mreD	Rod shape determining protein mreD
mreB	Rod shape determining protein mreB
**Secretary pathways**	
*secF*	Protein export membrane protein F
*virD4*	Type IV secretory pathway, VirD4 components
*Gd*	NAD-specific glutamate dehydrogenase
**Stress responsive pathways hways**	
*mlp5d*	Mannitol 1-phosphate 5 dehydrogenase
*p5cr*	Pyrroline-5-carboxylate reductase
*bgiG*	Mannitol operon activator, BglG family
**Carotenoid biosynthesis pathway esis pathway**	
*ps*	Phytoene synthase
*pd*	Phytoene desaturase
**Indole acetic acid biosynthesis pathway osynthesis pathway**	
*tsa*	Tryptophan synthase α chain
*tsb*	Tryptophan synthase β chain
*i3gps*	Indole-3-glycerol phosphate synthase


Real-time PCR conditions were; 95°C for 10 min, followed by 40 cycles of denaturation for 15 s at 95°C, and each annealing/elongation was performed for 1 min at 60°C. For each gene studied, the threshold (C_t_) value was normalized against the C_t_ for 16S rRNA conserved gene of *E. profundum* PHM11 that was used as the constitutive reference transcript (Rocha et al., 2015). The relative quantification of each of target gene with respect to the reference gene was calculated using below given mathematical model (Pfaffl, 2001).

$$Ratio(R)=\frac{{\left(E_{t\,\arg et}\right)}^{{\Delta ct}_{t\,\arg et}\left(control-sample\right)}}{{\left(E_{ref}\right)}^{{\Delta Ct}_{ref}\left(control-sample\right)}}$$

The relative expression ratio (*R*) of a target gene was obtained by calculating the deviation of real-time PCR efficiencies (*E*) and crossing point difference (*Ct*) of unknown versus control, and expressed in comparison to the reference 16S rRNA gene. Control *E. profundum* PHM11 cells grown in non-saline conditions were used as the calibrator. Later, relative expression ratios (*R*) values of each gene from non-saline control, 100 and 1500 mM treatments were compared statistically to get the significant differences in their expression patterns StepOnePlus^TM^ (Applied Biosystems) Real-Time PCR System was used to record and to analyze the fluorescent signal intensities.

### Cluster and Principal Component Analysis (PCA)

To get the impact of salinity on physiology and growth of PHM11, cluster and PCA analysis of various data including contents of IAA and proline, biomass and carotenoids yields, and growth kinetics data was performed using PAST 2.0.

### Statistical Analyses

Various experimental results obtained from the physiological and molecular studies such as carotenoids, IAA, beta-carotene, L-proline contents and biomass yields were analyzed statistically through two-way ANOVA with Bonferroni posttests using GraphPad Prism version 5.0 for Windows, GraphPad Software, San Diego, CA, United States. Non-linear logistic regression equation was generated by SigmaPlot 11.0. PCA and cluster analysis was performed in PAST 2.0.

## Results

### Bacterial Strain, Salinity Treatments and Sample Collections

The partially sequenced 16S rRNA gene of PHM11 has been submitted to GenBank under the accession number KF732974. Based on the obtained BLASTn analysis, indent score, BLAST score, ‘E’ value, and query coverage for 16S rRNA gene, bacterium PHM11 was identified as *Exiguobacterium profundum* PHM11. Initially, the logistic non-linear regression equation was developed to determine the viable cell density of *E. profundum* PHM11 by considering the cell counts through hemocytometer and corresponding OD values at 600 nm. Based on the mathematical observations, it was concluded that one unit absorbance at 600 nm is equal to 2.5 × 10^9^ cells mL^-1^ (*y* = 2.50057e+009; *R* = 0.973) for PHM11. For salinity stress experiments, 2.5 × 10^9^ cells of PHM11 were inoculated in nutrient broth (NB) alone, and with supplementation of 100 and 1500 mM sodium chloride salt, and grown for 72 h, until it reached to initial stationary phase. Fully grown, non-dividing cells of PHM11 were collected and total biomass yields were measured which was later used for extracting the total carotenoids, determining the IAA and L-proline contents, and purifying beta-carotene and getting the expressed RNA which alternatively was used in deciphering the salt-induced consequences affecting the bacterial physiology by changing the expression profiles of key genes of some regulatory pathways related to the plant growth promotion and stress tolerance.

### Salt Stress Affected the Growth Rates and Biomass Yields of *E. profundum* PHM11

**FIGURE 1:**
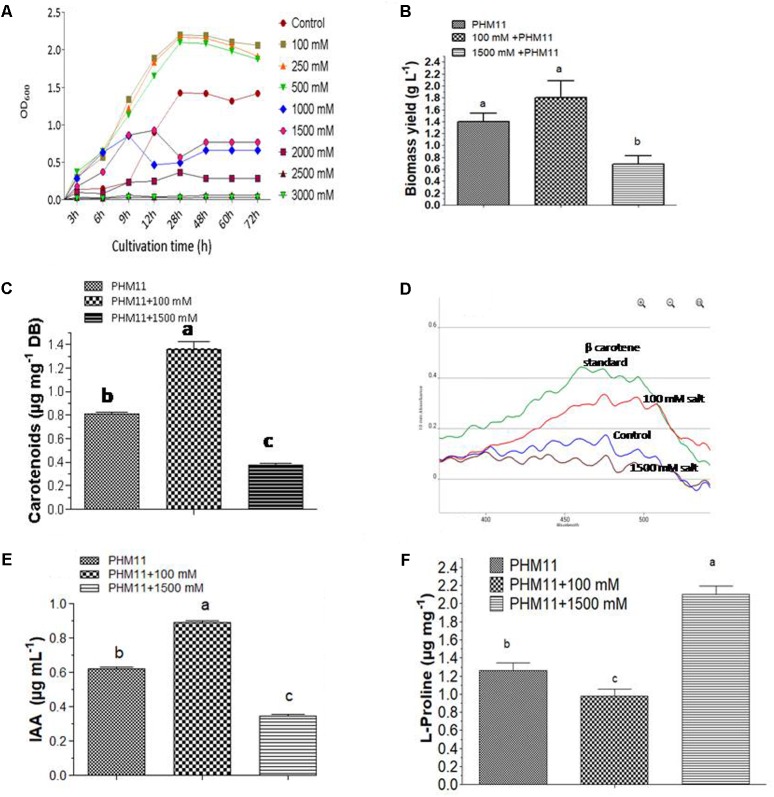
Growth curves and metabolite production by *E. profundum* PHM11 under different regimes of salinity **(A)** growth rates **(B)** biomass production **(C)** total carotenoid production **(D)** beta-carotene spectrophotometric profiles **(E)** indole acetic acid production **(F)** L-proline production. The error bars shown are ± standard deviation (SD) from the mean. All the experiments were performed in three replications (*n* = 3). ‘a’ stands for highest variation, followed by ‘b’ and lowest by ‘c’ from the experimental control.

During cultivation, we regularly monitored the growth rates of PHM11 under various experimental conditions. In comparison to experimental non-saline control cells, growth rates of 100, 250, and 500 mM salt-treated cells were found to be comparatively higher; however, with further increase in salt concentrations at higher doses, growth rates were started retarding and completely ceased at 2500 mM. Therefore, an important thing noticed was the increasing salt concentrations up to certain limits is beneficial in promoting the growth of this organism; yet, a further increase in the concentrations beyond 500 mM affects its growth negatively. Based on the observations, 100 and 1500 mM salt regimes were selected as optimal and minimal performance limits for *E. profundum* PHM11. In the next round of experiments, only 100 and 1500 mM salt regimes were provided to PHM 11 cells and results were compared with non-saline control. Not only growth rates, but also the morphological appearances of colonies were highly affected by 1500 mM salt regime. The color of colonies was slightly light orange at 1500 mM salinity when compared with 100 mM and non-saline control colonies. Stoichiometric growth kinetics parameters are given in **Table 2**. Growth curves of PHM11 under different experimental conditions are shown in **Figure 1A**. When 1500 mM treated culture was compared with control PHM11, statistically significant difference was recorded in the biomass yields. Yet, biomass yield of 100 mM treated culture was much higher than control PHM11, it was not found statistically significant at 5% probability. Maximum biomass yield of 1.86 g was obtained from the 100 mM treated cells, followed by control (1.40 g) and 1500 mM salt-treated cells (0.68 g), respectively (**Figure 1B**). Therefore, 100 mM salt-stress-induced the growth rate and biomass yield of *E. profundum* PHM11, however, biomass yield was not found statistically significant. In contrary, growth rate and biomass yield was highly retarded at the highest dose of 1500 mM. Statistical analysis with two-way ANOVA and Bonferroni posttests showed significant difference in the mean value of biomass yield at 95% confidence level (*p* ≤ 0.0006), and accounted 91.48% of total variance with Fisher value; *F* = 32.21, at the degree of freedoms; DFn = 2, and DFn = 6 for 1500 mM treated culture. As a result, maximum salinity concentration of 1500 mM severely affected the biomass yields of *E. profundum* PHM11.

**Table 2:** Stoichiometric growth kinetics of *E. profundum* PHM11 under different salt concentrations.

Salt concentrations	Number of generations (n)	Mean generation time (g) hour	Specific growth rate (μ) h^-1^	Maximum specific growth rate (μ_max_) (Cells mL^-1^h^-1^)
Non-saline control	6.6 ± 0.1	4.82 ± 0.01	0.062 ± 0.001	4.88 × 10^7^± 0.02
100 mM salt	6.8 ± 0.2	4.68 ± 0.01	0.044 ± 0.001	5.67 × 10^7^± 0.07
250 mM salt	6.7 ± 0.1	4.75 ± 0.01	0.063 ± 0.003	7.61 × 10^7^± 0.07
500 mM salt	6.7 ± 0.1	4.78 ± 0.05	0.063 ± 0.001	7.39 × 10^7^± 0.07
1000 mM salt	5.4 ± 0.2	5.92 ± 0.02	0.050 ± 0.002	1.27 × 10^7^± 0.09
1500 mM salt	5.5 ± 0.3	6.32 ± 0.02	0.047 ± 0.002	1.79 × 10^7^± 0.07


*For each data mean ± SD (standard deviation) values are shown. All the values were calculated from the average of three replications (*n* = 3).*

Analysis of stoichiometric growth kinetics data obtained from the various treatments showed that mild salt-stress enhanced the growth rate as depicted from the increased number of generations, specific and maximum specific growth rates, and decreased mean generation times of 100, 250, and 500 mM salt-affected cells of PHM11. In contrary, higher doses of 1000 and 1500 mM negatively affected the growth of PHM11. Detailed growth kinetics observations are provided in **Table 2**.

### Mild Salinity Stress Promoted the Carotenoids and IAA Production in *E. profundum* PHM11

Total carotenoid contents were measured from the control and salt-treated cells of the halotolerant PHM11 bacterium. Carotenoids concentration was found to be comparatively higher in 100 mM salinity affected cells, followed by non-saline control cells and 1500 mM salt-treated cells respectively. Corresponding values of carotenoids obtained for the non-saline control, 100 mM and 1500 mM salt-affected cells were 0.808, 1.362, and 0.376 μg mg^-1^ of biomass (**Figure 1C**). Results suggested that 100 mM and 1500 salt treatments affected the carotenoid production. 100 mM salt-affected PHM11 cells produced 168.48% higher carotenoids than non-saline control culture. On the other hand, 1500 mM salt-treated PHM11 cells could produce only 46.53% of non-saline control cells. Therefore, 100 mM salinity promoted the growth and carotenoids production of *E. profundum* PHM11. In contrary, highest dose of 1500 mM salt negatively affected the biomass yields and total carotenoid production. Therefore, mild salt stress promoted the growth and carotenoids production of this halotolerant organism while severe salt stress highly diminished the yields. Statistical analyses with two-way ANOVA and Bonferroni posttest showed 99.51% of the total variance and significant differences at 95% confidence level (*p* ≤ 0.05) in the mean values of carotenoids content of *E. profundum* PHM11 cells at different treatments with Fisher value; *F* = 603.84, and the degree of freedom; DFn = 2, DFd = 6. Absorption spectra of beta- carotene, purified from the equal biomass of different salt-treated cells of *E. profundum* PHM11 are shown in **Figure 1D**.

To evaluate that how the salinity can affect the plant growth promotion potential of this halotolerant organism, the concentration of phytohormone IAA was measured from the non-saline control, 100 and 1500 mM salt-treated cultures of PHM11. IAA concentration was maximally produced at 100 mM salinity, followed by non-saline control and 1500 mM treated cultures respectively. Corresponding values of total IAA content for non-saline control, 100 and 1500 mM salt-affected cells were 0.619, 0.819, and 0.346 μg mL^-1^ (**Figure 1E**). Therefore, 100 and 1500 mM salt treatments highly affected the IAA production. 100 mM salt-affected culture could produce 132.31% higher IAA than non-saline control organism. On the other hand, 1500 mM salt-treated culture of PHM11 could produce only 42.24% of non-saline control. Therefore, 100 mM salinity promoted the production of IAA by *E. profundum* PHM11 cells. However, the highest dose of 1500 mM salinity negatively affected the IAA production. Therefore, at 100 mM salt stress we could be able to get more IAA. Statistical analysis with two-way ANOVA and Bonferroni posttest showed 99.84% of the total variance and significant differences at 95% confidence level (*p* ≤ 0.05) in the mean values of IAA content of *E. profundum* PHM11 cells at different treatments with Fisher value; *F* = 3793.60, and the degree of freedom; DFn = 2, DFd = 12.

### Under Salinity L-Proline Biosynthesis Was Increased in Halotolerant *E. profundum* PHM11 Cells

Since L-proline is considered as a stress biomarker to define the level and impact of stress on cellular physiology of organism; therefore, we quantified the concentrations of L-proline from non-saline control, 100 mM and 1500 mM salt-affected cells of PHM11. At 1500 mM of salinity, L-proline content was highest while 100 mM salt-affected cells produced its least amounts. Corresponding quantified values of L-proline from non-saline control, 100 and 1500 mM salinity affected cells of PHM11 were 1.26, 0.976, and 2.1 μg mg^-1^ of biomass respectively (**Figure 1F**). Therefore, when cells were under severe stress condition, L-proline accumulated maximally in PHM11 cells to protect them from salinity induced consequences that negatively affected its overall cellular physiology. Two-way ANOVA and Bonferroni posttest showed 97.86% of the total variance and significant differences at 95% confidence level (*p* ≤ 0.05) in L-proline contents of *E. profundum* PHM11 cells at different treatments with Fisher value; *F* = 274.26, and degree of freedom; DFn = 2, DFd = 12.

### Salt Stress Affected the Expression of Different Metabolic Pathways

Salt treated *E. profundum* PHM11 cells were used to study the expression of genes related to salt-stress responsive pathways, transcription and translation, protein folding, structural adaptations, and carotenoid biosynthetic pathway. A total of 22 genes (**Table 1**) were analyzed for the changes in their expression patterns under salt stress. Genes participating valuable roles in direct or indirect salinity stress mitigation, providing structural adaptations and affecting the important metabolites production under stress were selected from *E. profundum* PHM11 genome. It was an approach to decipher the roles of genes in affecting the physiology of PHM11 under salinity stress.

### Genes Related to Structural Adaptations

**FIGURE 2:**
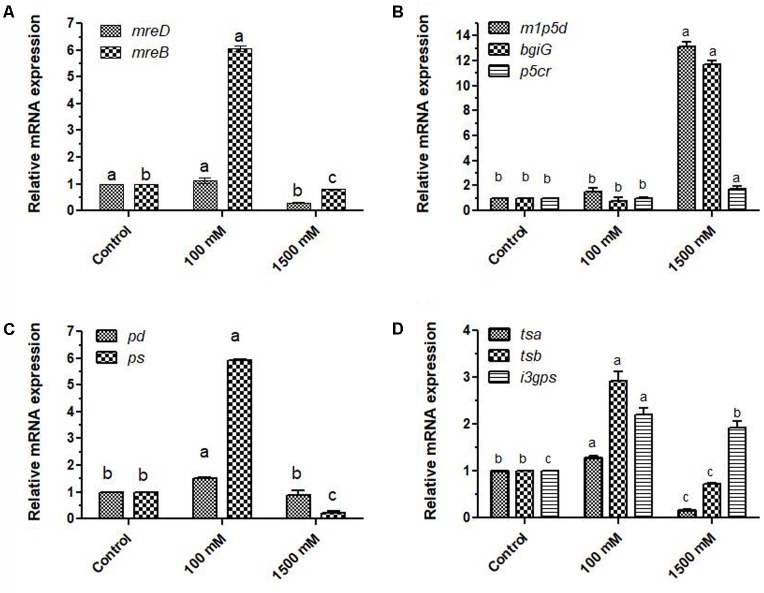
Relative fold changes in the expression profiles of genes related to shape determination, stress mitigation, carotenoid production, plant growth promotion under salinity **(A)** relative fold changes in the expression of shape determining genes; *mreB* and *mreD*, **(B)** relative fold changes in the expression of L-proline (pyrroline-5-carboxylate reductase; *p5cr*) and mannitol pathway genes (mannitol 1-phosphate 5 dehydrogenase; *m1p5d*, mannitol operon activator BglG family; *bgiG*), **(C)** relative fold changes in the expression of carotenoid biosynthesis related genes (phytoene desaturase; *pd*, phytoene synthase; *ps*), **(D)** relative fold changes in the expression of shikimate pathways genes related to the production of tryptophan and indole acetic acid (tryptophan synthase α chain; *tsα*, tryptophan synthase β chain; *tsβ* and indole-3-glycerol phosphate synthase; *i3gps*). The error bars shown are ± standard deviation (SD) from the mean. All the experiments were performed in three replications (*n* = 3). ‘a’ stands for highest variation, followed by ‘b’ and lowest by ‘c’ from the experimental control.

Since micrographs of control and salt treated cells showed statistically significant variation in their diameters (Supplementary Figure 2), expressions of rod-shape determining protein *mreD* and *mreB* were analyzed to understand how these structural genes get affected by salinity with 100 and 1500 mM salt concentrations. At mild stress of 100 mM salinity, their expression got increased by 1.12 and 6.07 folds, while 1500 mM salinity reduced the expression by 0.30 and 0.80 folds (**Figure 2A**) which is also being justified from the micrographs. The 100 mM salt-affected PHM11 cells have slightly larger length as compared to non-saline control and 1500 mM salt-affected PHM11 cells (Supplementary Figure 2).

### Stress Responsive Pathways

Proline and mannitol are considered as stress-protecting compounds. In general, their levels get increased under different abiotic stress. Expression of key genes of mannitol biosynthesis (mannitol 1-phosphate 5 dehydrogenase; *m1p5d*, mannitol operon activator, BglG family; *bgiG*) and proline biosynthesis (pyrroline-5-carboxylate reductase; *p5cr*) pathways were highly affected by salinity. In 100 mM salt-treated PHM11 cells, expression of *m1p5d* and *p5cr* were 1.5 and 1.01 fold higher (**Figure 2B**), while *bgiG* showed only 0.77 fold expression when compared to non-saline control PHM11 cells. In contrast, 1500 mM salt-treated cells of *E. profundum* PHM11 showed 13.1, 1.7, and 11.7 fold increased expression of *m1p5d, p5cr* and *bgiG* genes, respectively. Therefore, under severe stress condition of 1500 mM salinity, our results suggested enhanced expression in the key regulatory genes of proline and mannitol biosynthesis pathways. Two-way ANOVA and Bonferroni posttests showed statistically significant (*p* ≤ 0.05) variations in the expression patterns of these genes with respect to non-saline control *E. profundum* PHM11 cells.

### Carotenoid Biosynthesis Pathway

In comparison to control PHM11 cells, expression of phytoene desaturase; *pd* in 1500 mM salt-affected cells was only 0.09 fold. Expression of phytoene synthase and phytoene desaturase genes were increased by 1.52 and 5.92 folds in 100 mM salt-treated *E. profundum* PHM11 cells (**Figure 2C**).

### Expression of Genes Related to Indole Acetic Acid Metabolism

Effect of salt stress on the production of Indole acetic acid was studied through elucidating the changes in gene expression profiles of IAA pathway genes; tryptophan synthase α chain; *tsα*, tryptophan synthase β chain; *tsβ* and indole-3-glycerol phosphate synthase; *i3gps* were analyzed (**Figure 2D**). The expression of *tsα, tsβ and i3gps* were reduced at 1500 mM dose of salinity. However, at 100 mM salinity, their expressions were increased with respect to non-saline control. Expression patterns of *tsα* and *tsβ* were highly affected than *i3gps* under severe salinity.

### Secretary Pathways

**FIGURE 3:**
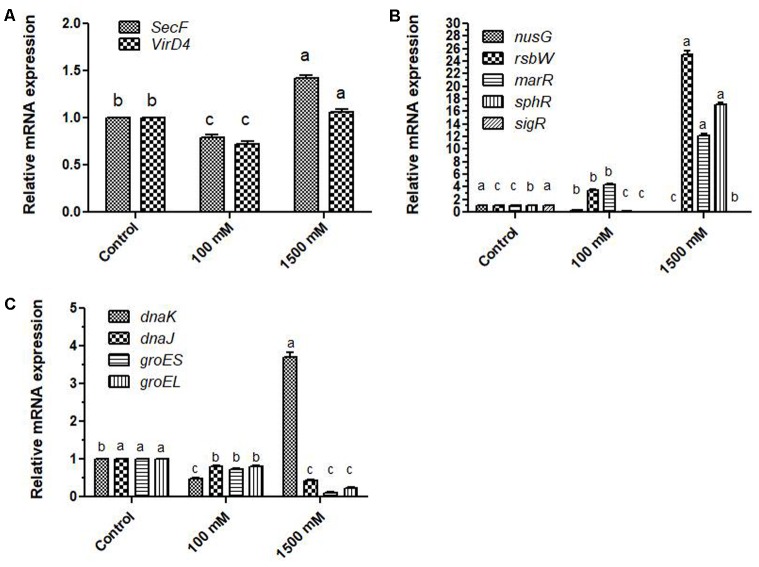
Relative fold changes in the expression profiles of genes related to secretion of metabolites, transcription and translation of regulatory proteins, and protein folding related chaperons and chaperonins under salinity **(A)** relative fold changes in the expression profiles of genes related to secretion Protein export membrane protein; *secF*, VirD4 components; *virD4*), **(B)** relative fold changes in the expression profiles of genes related to transcription and translation (transcription anti-termination protein; *nusG*, serine-protein kinase; *rsbW*, transcriptional regulator; *marR*, phosphate regulon transcriptional regulatory protein PhoB; *sphR*, and RNA polymerase sigma factor SigV; *sigR*), **(C)** relative fold changes in the expression profiles of genes related to protein folding (chaperon protein DnaK; *dnaK*, chaperon protein DnaJ; *dnaJ*, heat shock protein 60 family chaperon GroES; *groES*, heat shock protein 60 family chaperon GroEL; *groEL*). ‘a’ stands for highest variation, followed by ‘b’ and lowest by ‘c’ from the experimental control.

To see that how the expression of genes related to the export of biomolecules across the membranes is affected, three genes; (1) protein export membrane protein F; *secF*, (2) type IV secretory pathway, VirD4 components; *virD4*, (3) NAD-specific glutamate dehydrogenase; *gd* were evaluated under 100 and 1500 mM salinity. Expression of *secF* and *virD4* was reduced at 100 mM regime; however, at 1500 mM expression of these genes got increased 1.41 and 1.06 folds. In contrast, expression of *gd* was highly affected at both 100 and 1500 mM doses of salinity, and 111.69 and 590.97 folds changes were observed respectively (**Figure 3A**). Two-way ANOVA and Bonferroni posttest showed highly significant variations in the expression pattern of *gd* at 95% probability (*p* ≤ 0.05).

### Expression of Proteins Fine-Tuning the Transcription and Translation of Key Genes

Among the prokaryotes, transcription factors are not well known; however, similar proteins that regulate the transcription and translation are known. To know that how the salinity can affect their expressions, five genes coding for regulatory proteins namely; (1) transcription anti-termination protein; *nusG*, (2) serine-protein kinase; *rsbW*, (3) transcriptional regulator; *marR*, (4) phosphate regulon transcriptional regulatory protein PhoB; *sphR*, and (5) RNA polymerase sigma factor SigV; *sigR* were analyzed under 100 and 1500 mM salinity. Expression of the *nusG* gene at 100 and 1500 mM salinity was only 0.33 and 0.006 folds in comparison to control cells while *sigR* expression was found to be reduced by 0.0059 and 0.082 folds respectively. On the other hand, *sphR* showed only 0.22 and 17.15 fold expression at 100 and 1500 mM salinity. In contrast, expression of *rsbW* and marR genes got enhanced by 3.45, 25.0 and 4.35 and 12.2 folds respectively in 100 and 1500 mM salt-affected cells of *E. profundum* PHM11. Expression profiles of different genes are shown in **Figure 3B**.

### Protein Folding

Expression of four genes related to protein folding namely (1) chaperon protein DnaK; *dnaK*, (2) chaperone protein DnaJ; *dnaJ*, (3) heat shock protein 60 family chaperon GroES; *groES*, (4) heat shock protein 60 family chaperon GroEL; *groEL* were analyzed under 100 and 1500 mM salinity. In comparison to non-saline control, expression of *dnaK* was only 0.48 folds at 100 mM while at 1500 mM, it was enhanced to 3.7 folds. Expression of other folding related genes was reduced at both 100 and 1500 mM salinity (**Figure 3C**).

### Multivariate Analysis

**FIGURE 4:**
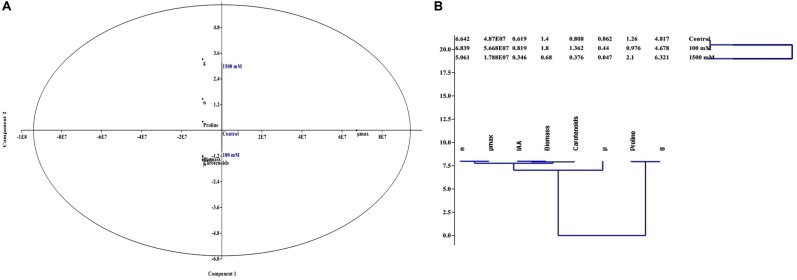
Principal component and cluster analysis showing that how the salinity affects the growth and physiology of *E. profundum* PHM11 **(A)** PCA plot **(B)** cluster analysis.

Considering the measured parameters related to the growth and physiology of PHM11 such as biomass, carotenoids, IAA, proline, number of generation, mean generation time, and specific growth rate under different regimes of salinity; a PCA plot was generated by putting all the traits into three different components. The results of PCA plots clearly indicated that 100 mM salinity highly increased the number of generation, biomass, IAA, and carotenoid production while proline and mean generation time were negatively affected. In contrast, 1500 mM salinity increased the production of proline and mean generation time while other evaluated parameters were negatively affected (**Figure 4A**). Results of cluster analysis further supported the PCA, and clarified that IAA, biomass and carotenoid production are directly related to the specific growth rate and number of generation of PHM11. Similarly, proline content and mean generation time are directly related with each other, as exemplified by the higher proline content and mean generation time of 1500 mM salt-affected bacterial cells (**Figure 4B**).

## Discussion

A number of halotolerant bacteria are well documented to tolerate varying levels of different abiotic stresses. *E. profundum* is known as a halotolerant organism. This organism is a vital source of useful carotenoids (Rodrigues et al., 2008; Shatila et al., 2013). To date, published literature has very little information about the mechanism that how the different strains of *Exiguobacterium* tolerates the salinity by modulating its inherent physiology and fine-tunes their gene expression profiles. A regular search for such novel halotolerant organisms that can have a number of plant growth promotion traits is under investigation globally. These organisms can be enriched to rhizosphere of agricultural crops to mitigate the salinity induced consequences in variety of plants. Such microbes can be used as an efficient tool to establish unique approaches against salinity-induced challenges in the plants, and to discover hitherto concealed pathways related to the salt tolerance. In the present study, efforts were made to decode the role of some key regulatory genes of a halotolerant bacteria *E. profundum* PHM11 under salinity, and possible mechanism behind the salt-tolerance by this organism was tried to be elucidated at some extent through correlating the expression patterns of these genes with the amounts of stress biomarkers such as proline, IAA, and carotenoids produced from the non-saline control and salt-treated PHM11 cells. By doing so, inferences could be drawn that how the changes in expression profiles are related to the production of stress tolerant metabolites. Nevertheless, realistic consequences, biochemical and molecular mechanisms underlying the salinity tolerance by *E. profundum* PHM11 are far away to be understood. To begin with this issue, halotolerant *E. profundum* PHM11 bacterium was used as model organism in this study to decipher those key genes, whose expressions were affected by salinity, and results were correlated with altered bacterial morphology, biomass productivity and physiology. Initially, salt tolerance of halotolerant bacteria *E. profundum* PHM11 was elucidated under varying regimes of salinity to tackle its realistic minimal and optimal salt-tolerance limits. Considering the evaluated ranges of salinity, i.e., 100, 250, 500, 1000, 1500, 2000, 2500, and 3000 mM; useful finding that up to a concentration of 500 mM, growth rate of this organism was increased, however, beyond that level of salinity, its growth was started retarding drastically. Therefore, 100 and 1500 mM salinity were considered to further test the growth rates, biomass yields, and expression of key genes related to the salt tolerance, production of stress protecting compounds, and growth promoter auxin, and altering the cell morphology. In addition, some useful anti-oxidant compounds such as beta-carotene were purified from this halotolerant bacterium. Moreover, molecular and biochemical responses of *E. profundum* PHM11 were investigated under salinity. Amongst salinity induced consequences; biomass yields, accumulation of L-proline, production of growth promoter; IAA, total carotenoids, beta-carotene, and rapid induction of abiotic stress responsive genes were analyzed.

The halotolerant bacterium *E. profundum* PHM11 can grow efficiently up to 500 mM salinity, as exemplified by the biomass yields of non-saline control and biomass recovered from the PHM11 cells affected with different salt concentrations. With respect to non-saline control cells of PHM11, cells affected with 100, 250, and 500 mM salinity showed increased growth rates; however, beyond this concentration growth rates started decreasing (Supplementary Figure 1). Biomass yields of 1.80, 1.72, 1.64, 1.1, and 0.68 g was obtained respectively for 100, 250, 500, 1000, and 1500 mM salt affected cells of *E. profundum* PHM11. As exemplified by the biomass yields of salt-affected cells of *E. profundum* PHM11, the growth of this halotolerant bacterium was highly slowed-down with increasing salinity. Biomass yield of 1.4 g was obtained for the control cells of *E. profundum* PHM11. Therefore, salt concentration up to 500 mM affected the biomass yields positively, however, beyond this limit growth and biomass yield was affected negatively. At 100, 250, and 500 mM salinity 128.5, 122.88, and 117.14% enhanced biomass yield was obtained. In contrary, with respect to control considered as 100%, at 1000 and 1500 mM salinity, only 78.57 and 48.57% biomass production was obtained from *E. profundum* PHM11 cells respectively. Therefore, higher salt regimes reduced the biomass yields. In a study, by Awata et al. (2015), it was demonstrated that mild salinity stress is helpful in inducing the bacterial growth rates and biomass yields, however, when the levels of salinity is increased it became dangerous for bacterial health, and drastically affected the overall productivity and physiology. They observed the decreased biomass yields with increased salinity in a marine anammox bacterium *Candidatus Scalindua* sp. (Awata et al., 2015).

Salinity directly affects the cell morphology of bacteria. Microscopic examinations of salt-treated and control cells showed an increase in diameter of PHM11 cells at 100 mM salinity with respect to non-saline control cells (Supplementary Figure 2). On the other hand, 1500 mM salinity significantly reduced the cell diameter. Not only that, but also dead cells were clearly seen in micrographs recorded for 1500 mM salinity affected PHM11 cells. It could be a probable reason behind altered biomass production of salt-treated PHM11 cells with respect to its non-saline control. Expression of rod-shape cellular architecture determining genes of *E. profundum* PHM11 got extremely influenced by different salt concentrations, as observed by the changes in the expression profiles of *mreD* and *mreB;* at 100 mM salinity, their expression got increased by 1.12 and 6.07 folds, while at 1500 mM salinity expression got reduced by 0.30 and 0.80 folds. Therefore, to maintain original rod -shape of *E. profundum* PHM11 cells, expression of these shapes determining genes were altered to maintain the original morphology of PHM11 cells. However, *mreB* gene was negatively affected by salinity, and it may be a cause to distort the shape of PHM11 cells. With transcriptomic approaches, we could be able to get the induction or suppression of rod shape determining genes under salinity. Present experimental findings, indicates that expression of shape-determining genes *mreD* and *mreB* got variably affected at different levels of salinity which alternatively influenced the diameter of cells and biomass yields.

In order to find out vigorous genes that determine the shape of bacteria, whose expressions are altered in saline soils or under saline experimental conditions, much progress has been made through modern approaches of transcriptomics. Given this progress, bacteriologists may inquire why they should care about the details of the concentrations of salts in sodic soils or in culture media. Yet, a number of theoretical issues on the impact of salinity affects on the inherent physiology of halotolerant bacteria, that could not be address without understanding the real changes in gene expression profiles under a particular saline environment for bacterial species, may be generated through studying their expression patterns under different regimes of salinity. Bacterial operon *mreBCD* is responsible for providing rod shape to different bacteria, it is highly regulated by environmental gradients, and completely absent in spherical cells (Kruse et al., 2005). All the *mreBCD*-encoded components are essential for maintenance of rod-shape, depletion in its any of component is responsible for septum placement, providing a spherical and enlarged cell that alternatively get lyses (Levin et al., 1992; Kruse et al., 2005). *mreB*, an actin-homologue, determines the cell shape in most of the rod-shaped bacteria (Figge et al., 2004). A mutation in *mreB* gene generated spherical cells in *Anabaena* sp. PCC 7120, which normally have rod-shaped cells (Hu et al., 2007). Primer extension studies also identified that three genes of *mreBCD* operon have individual transcriptional initiation sites and unique promoters, and most of the time *mreB* is expressed monocistronically (Wachi et al., 2006). Collectively, proteins coded by *mreB, mreC, and mreD* genes form bacterial cytoskeleton by creating the actin-like cables lying beneath the cell membranes (Salje et al., 2011).

Osmoadaptation is a common phenomenon in halotolerant organisms; it basically involve two steps (1) salt in cytoplasm mechanism; in this halotolerant bacteria creates osmotic equilibrium through maintaining the cytoplasmic salt concentration like to that of the bathing solution, it requires extensive structural modifications (2) osmoprotectant accumulation; when first response does not work then bacteria starts second mechanism to protect themselves from the salinity (Sleator and Hill, 2002). Salt tolerance limits of most of the prokaryotes are highly variable; yet, beyond 500 mM salt, a majority of bacteria start to accumulate glutamate, glycine betaine, proline, mannitol, ectoine, and carnitine etc. (Sevin et al., 2016).

In the present study; we quantified the L-proline in non-saline control, 100 and 1500 mM salt-treated cells of *E. profundum* PHM11. In 1500 mM salt affected cells proline content was highest; however, in 100 mM salt-affected cells it was found to be lowest. Since growth studies showed that 100 mM salinity enhances the growth rates; consequently, comparatively to control cells, 100 mM salinity affected cells had better health which may be a cause for detection of the lowest concentrations of L-proline. In 100 and 1500 mM salt-treated PHM11 cells, expression pattern of *p5cr, a* key regulatory gene of proline biosynthesis was found to be 1.01 and 1.7 folds respectively. Our results showed non-significant changes in expression profiles at 100 mM dose; however, at 1500 mM dose its expression got enhanced by 1.7 folds which indicate the higher accumulation of proline under salinity. Therefore, as depicted from the gene-expression and L-proline data, it is clear that *p5cr* significantly enhanced the production of L-proline under 1500 mM salinity. It was also justified by the quantified values of L-proline from control, 100 and 1500 salt-affected cells as 1.26, 0.976, and 2.1 μg mg^-1^ respectively. Pyrooline -5-carboxylase synthase (*p5cs*) and Pyrooline -5-carboxylase reductase (*p5cr*) are well known enzymes regulating the proline biosynthesis under various stresses (Perez-Arellano et al., 2010). So, *p5cr* gene expression highly regulated the accumulation of proline in different salt-treated cells. In a study on pshychrophilic diatom *Fragilariopsis cylindrus*, 2.5 fold increased up-regulation of *p5cr* gene significantly improved the salt tolerance and photosynthetic efficiency (Krell et al., 2007). The up-regulated expression of *p5cr* gene has been reported to manage the proline metabolism in water-logged and drought stressed *Arabidopsis* plant also (Ghosh et al., 2017). Proline is reported as a multiple abiotic and biotic stress alleviator molecules in various plans and microbes (Willet and Burton, 2002; Liang et al., 2013). Here, we could be able to get the gene regulatory mechanism for the increased production of proline under 1500 mM salinity.

Besides, proline metabolic pathway, we also investigated the expression of two genes of mannitol biosynthesis pathway; *m1p5d* and *bgiG* at 100 and 1500 mM salinity. With respect to control; 1.5 and 13.1 fold for *m1p5d*, and 0.77 and 11.7 fold altered expressions for *bgiG* was obtained respectively under 100 and 1500 mM salinity. These results directly indicate the differential gene expressions of these genes at low and high salt concentrations. Salinity reduces microbial biomass mainly because the osmotic stress results in drying and lysis of cells. Proline, glycine betaine and mannitol are the main organic osmo-protecting compounds functioning as osmolytes accumulated by salinity tolerant microbes (Csonka, 1989). Therefore, by fine-tuning the expression profiles, these genes helped PHM11 cells in accumulating the higher level of osmo-protectants. Our results suggested that in response to stress, proline accumulation rates of *E. profundum* PHM11 exposed to salt stress were highly increased; therefore, cells accumulated its plentiful amounts which alternatively helped in maintaining the osmotic potential of bacterial cells by facilitating the salt in cytoplasm mechanism to maintain the osmotic pressure and improved the salt-tolerance. Mannitol 1-phosphate 5 dehydrogenase was found to regulate the mannitol biosynthesis in red algae *Caloglossa continua* under 200 mM salinity (Iwamoto et al., 2003). *Zea mays* and *Peanut* plants were transformed by bacterial mannitol 1-phosphate 5 dehydrogenase transgene to enhance their salinity tolerance (Sticklen et al., 2013) and drought tolerance (Bhauso et al., 2014) respectively. Pyrroline-5-carboxylate (P5C), a key intermediate in proline metabolism is increased under both biotic and abiotic stresses. P5C is synthesized from the glutamate through enzyme pyrroline-5-carboxylate synthase (Hu et al., 1992), subsequently converted to proline through pyrroline-5-carboxylate reductase; P5CR. In *Arabidopsis thaliana*, it was found to actively manage the osmoregulation through fine-tuning its mRNA levels in ripening seeds (Verbruggen et al., 1993). Efforts have been made to improve the stress tolerance of plants by introducing this gene from bacterial systems (Perez-Arellano et al., 2010).

Carotenoids are well-known compounds in bacteria that help in improving the salt tolerance. In order to see its production in stressed PHM11 cells, total carotenoids were extracted from the cells treated with 100 and 1500 mM salt, and expression of two genes; phytoene desaturase (*pd*) and phytoene synthase (*ps*) related to carotenoid biosynthesis pathway were analyzed. In comparison to control cells, total carotenoids production of 100 mM affected PHM 11 cells was 168% higher while cells treated with 1500 mM salt could produce only 46% with respect to non-saline control cells. Transcriptomic studies showed that expression of phytoene desaturase at 1500 mM salinity was negligible in comparison to control. In contrast, 100 mM salinity increased the yields of total carotenoids in *E. profundum* PHM11 cells through enhancing the expression of *ps* and *pd* genes by 1.52 and 5.92 folds respectively. In a study, alternative transcripts of carotenogenic genes *crtYB*; coding for phytoene synthase and *crtI*; coding for phytoene desaturase were differentially expressed in *Xanthophyllomyces dendrorhous* yeast grown on non-fermentable; succinate and a fermentable carbon source; glucose (Wozniak et al., 2011). Expression of carotenogenic genes; phytoene synthase and phytoene desaturase in green algae *Haematococcus pluvialis*, under combined light and salinity stress was enhanced, as a result, total carotenoid production enhanced to 32.0 mg g^-1^ of dry biomass (Vidhyavathi et al., 2008).

Auxins are phytochemicals that promotes plant growth, required in very minute amounts for helatier plant growth. Some bacteria such as *Bacillus, Pseudomonas, Rhizobium*, etc. have inherent potential to produce indole 3-acetic acid, by doing so they help surrounding plants to grow faster and healthier. *E. profundum* PHM11 produces IAA in considerably good amounts; approximately 0.619 μg mL^-1^. When its cells were exposed to 100 and 1500 mM salinity, their IAA content was highly affected, and at 100 mM salinity, it was enhanced by 132%. Expression profiles of three key genes of IAA biosynthetic pathway; *tsα, tsβ* and *i3gps* genes showed enhanced expression at 100 mM salinity; however, at 1500 mM salinity their expressions were reduced. Therefore, with enhanced IAA contents at 100 mM salinity expression of these regulatory genes were also enhanced. Furthermore, with a decrease in the IAA content at 1500 mM salinity, expressions of these three genes were also reduced. In an experiment, a number of *Pseudomonas* strains were evaluated for IAA production under varying regimes of salinity. IAA production was enhanced in all the strains up to 150 mM, beyond that concentration of salt, IAA production was highly decreased (Deshwal and Kumar, 2013). In a study, exogenous application of 1 mM phytohormone indole-3-acetic acid (IAA) stimulated the biofilm formation and EPS production that alternatively helped *Bradyrhizobium japonicum* in tolerating the stress. This dose was sufficient to modulate the expression profiles of approximate 1,323 genes in *B. japonicum* (Donati et al., 2013). In another study, indole acetic acid enhances the tolerance of tomato plants against heat stress by protecting against the DNA damage (Siddiqui et al., 2017). Therese, *tsα, tsβ* and *i3gps* genes are directly involved in regulating the IAA production in PHM11. Since, stress directly affects the membranes; therefore transport across the membrane is highly modulated. Therefore, studying the expression profiles of related genes is highly desirable in case of PHM11. Import and export of different metabolites under salt-stress is commonly affected. Expression patterns of three genes *secF, virD4*, and *gd* related to export and import of metabolites and proteins from cell membrane was highly affected. *secF* and *virD4* showed reduced expression at 100 mM salinity, while at 1500 mM expression was enhanced by 1.41 and 1.06 folds respectively. NAD-specific glutamate dehydrogenase; *gd* expression was enhanced by 111.6 and 590.9 folds respectively at 100 and 1500 mM salinity. The protein *virD4* is a part of type IV secretion systems in bacteria mediating the transport of protein and/or DNA (Redzej et al., 2017). The virD4 complex helps in spread of the antibiotic resistance genes among bacterial populations through secretion of DNA (Redzej et al., 2017). Therefore, it was necessary to study the role of salinity in altering its gene expression patterns.

Some common transcription and translation related proteins were also analyzed through transcriptomics approaches, and under two regimes of salinity, we got differential expression patterns. For instance, transcription anti-termination protein gene *nusG* was slightly reduced at 100 mM salinity. It has been reported that in rapid budding bacterial cells plateau phase comes earlier, therefore, transcription gets terminated for most of the genes. At 100 mM salinity we got the rapid growth of the PHM11 cells (**Figure 1A**). Similarly, expression of SigV protein, coding for RNA polymerase sigma factor was also reduced at both 100 and 1500 salt regimes. In contrast, expression of serine-protein kinase; *rsbW* and transcriptional regulator; *marR* genes were enhanced in salt-affected cells of *E. profundum* PHM1. In a study, expression profiles of *rsbW and SigV* genes were found to be affected by cold stress in *Exiguobacterium* (Dall’Agnol et al., 2014). How salinity affects the functioning at transcription level, to know this we selected *rsbW gene for expression studies. Sigma B* is a well-known secondary sigma factor of *Bacillus subtilis*. RNA polymerase having sigma B is able to transcribe the subset of genes that are expressed after stress. Three genes; *rsbV, rsbW*, and *rsbX* are co-transcribed along with the sigma B structural gene (sigB), and regulates *sigB*-dependent gene expression. *RsbW* is the main inhibitor of this system; it inhibits the formation of a sigma B-containing RNA polymerase holoenzyme.

Expression profiles of genes related to protein folding: *dnaK*, DnaJ; *dnaJ, groES*, and *groEL* were variably affected under different experimental conditions. Expect *dnaK* from 1500 mM treated PHM11 cells, expression patterns of other three genes; *dnaj, groES*, and *groEL* were reduced at different extent. It directly, shows that folding of proteins got affected by the salinity (**Figure 3C**). Moreover, expression patterns of these genes under salinity were highly modulated to keep their transcription at optimal levels.

## Conclusion

Salinity is a critical factor determining the life distribution under extreme environments. The combined approaches of microbial stress physiology and transcriptomics used in the present study allowed us to verify the molecular mechanisms of salinity adaptation chiefly manifested as changes in the gene expression profiles, and secondarily as differential growth rates, biomass yields, altered physiology and morphology. Transcriptomic approaches generated a reasonable coverage, which was also endorsed through altered biomass yields, L-proline, β carotene and IAA production, and cell elongation. Our findings suggest that *E. profundum* PHM11 exhibits a stress regulatory network governing the gene expression which alternatively ensures its survival and growth in varying saline environments, as a halo-tolerant organism. Selected genes were found to be differentially expressed among the two growth saline conditions, 100 and 1500 mM. It was related to maintain the integrity of bio-molecules and the physiological process, to preserve the cell architecture and function. Further integrated analyses are required to understand the gene regulation of the salinity sensitive pathways, helping to decipher the lifestyle of the *E. profundum* PHM11 isolated from the barren land of Varanasi, India.

## Author Contributions

AlKSr, AKSa, and VP designed the experiments. AlKSr and VP drafted this manuscript. VP and RS performed all the physiological and transcriptomic experiments. AS isolated *E. profundum* PHM11 and characterized it with RS. AnKSr performed 16S rRNA sequencing. SS assisted VP in bacterial cultivation. VP and RS performed all the statistical analysis. AK helped in transcriptomic studies. AlKSr, and AKSa reviewed this manuscript. HC, KP, and PK were Co-PIs of the project (AMAAS-Genomics).

## Conflict of Interest Statement

The authors declare that the research was conducted in the absence of any commercial or financial relationships that could be construed as a potential conflict of interest.
