# Supplementary material for: Halotolerant Exiguobacterium profundum PHM11 Tolerate Salinity by Accumulating L-Proline and Fine-Tuning Gene Expression Profiles of Related Metabolic Pathways
Source: Front Microbiol. 2018 Mar 12;9:423. doi: 10.3389/fmicb.2018.00423 (PMC5890156; doi:10.3389/fmicb.2018.00423)
Supplement: Supplementary file 1 [file Table_1.DOC]

**Supplementary information**

Halotolerant *Exiguobacterium profundum* PHM11 tolerate salinity by accumulating stress protecting compounds and fine-tuning gene expression profiles of related metabolic pathways

Vikas Kumar Patel, Ruchi Srivastava, Anjney Sharma, Anchal Kumar Srivastava, Savita Singh, Alok Kumar Srivastava*, P. L. Kashyap, Hillol Chakdar, K. Pandiyan, Alok Kalra, Anil Kumar Saxena

**Supplementary figure 1:** Evaluation of salt-tolerance limits of halotolerant bacterium *E. profundum* PHM11

**
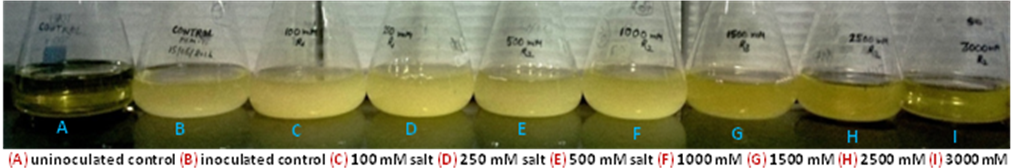
**

**Supplementary Figure 2:** Micrographs recorded at 30 µm bar scale for non-saline control, 100 and 1500 mM salt treated PHM11 cells.


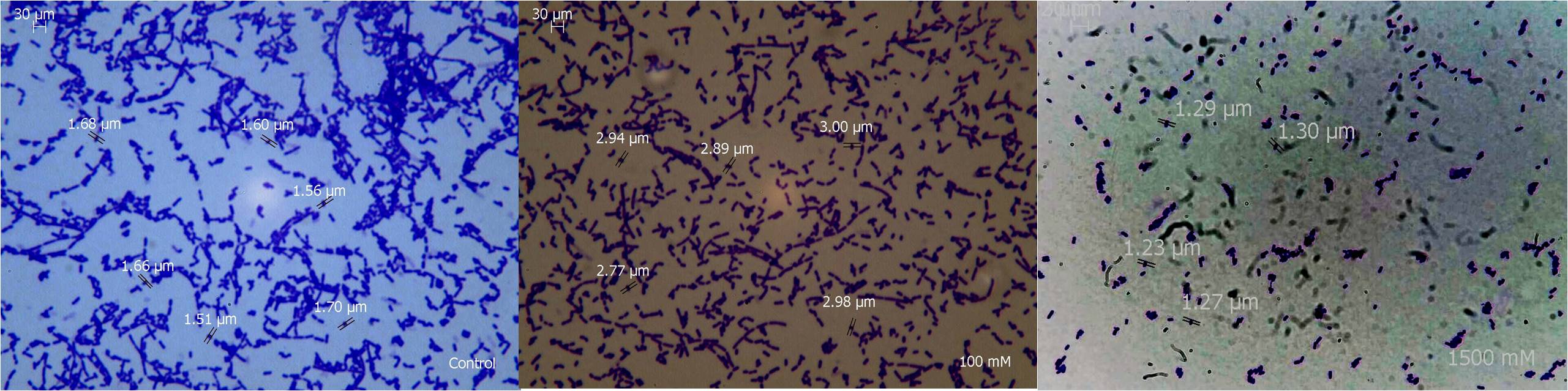


**Supplementary Table 1 Real-time PCR primers used for studying the expression profiles of key genes of some metabolic pathways of *E. profundum* PHM11.**

| ***Gene*** | **Forward primer** | **Reverse primer** | **Amplicon size** |
| --- | --- | --- | --- |
| *p5cr* | ATGAAAGGCTCCGCTGAGTTACTG | CTGAAGCCATCACGTCAGCAAGA | 142 |
| *marR* | ATGGACGTCGTCTATCCGCAACAT | TTTGCCCGCTGCGTTATAACC | 123 |
| *SigR* | ATGAAGAAGAGACAAAATGTGATT | TGAAGAGAATCCATCATATCAGC | 116 |
| *SphR* | ATGATTCGAGAAAAGTTAGGCGA | TCCGGAACGCTCCTCGGT | 99 |
| *rsbW* | ACGTTCGCAGATGATGTGAAGCG | CTTTATTGATCGAGACATCGTCCATT | 130 |
| *nusG* | ATGACTGACGATTCATGGTATGTT | ATCTTTCTTCTCGATGAGTCCC | 150 |
| hspGroEL | ATGAAGCGACGGGTGTCAAACT | ACCGACACCGACTGACTCGTTCT | 129 |
| hspGroES | ATGTTAAAACCACTAGGCGATCGTGT | GCGAAAGAAAAGCCGCAACAAG | 121 |
| dnaK | ATGATTCATCGTCAACTGCTTG | CGTCACGATACCTGACTCGAA | 130 |
| *dnaJ* | AGTGTTCGTGGTGGTGCAAATGGTGA | TTCGACACCGGACTCTCCGCTAAT | 129 |
| *mreB* | ATGGACCGCGGAATCGTCTT | CAGTCCCGACCGCTACGCA | 130 |
| *mreD* | ATGATTCTCGTTTTCACCTTTG | TTGACGAGCATCGTCCCTG | 135 |
| *sec F* | ATGGAGGTCTATGAGTCGATCATCAACA | TGCCCCGAATACATACAAGGCAA | 114 |
| *virD4* | ATGGGACTCAAAGAGGGCGAGAT | GTCGAAGTCGTCCGCCAGGTAT | 147 |
| *gd* | ATGTACGATGCAGGGGCGAA | GTGTTTTTAAATAATGTCGAAATCGTA | 137 |
| *bgiG* | ATGCAGATTCGGGTGCATA | ACTGTACTCGTCTCTGGTAGCG | 146 |
| *m1p5d* | ATGGATAACGGCATCGAGCC | GATGCCGTGTTCTTCGATCGAC | 120 |
| *ps* | ATGTTACACCAACGAGAAGTGACCC | TCGACTCGCGAGTGGATACAGAT | 122 |
| *pd* | ATGGATAAAATTTTTGAGAAACACGA | CGGAATGTGTGGCAAGATTTTGA | 129 |
| *tsa* | ATGACCTACTTGAATCCAGTGTT | ATTGACATCTGCGAACAACTGA | 135 |
| *tsb* | ATGGGGATGTTCTATCCGTTTCTA | GAGCGACCCATGTAAGACACCG | 138 |
| *i3gps* | ATGAAACGCTATCCTGAGGTCACATT | GGTCGGGTCCTCTTCACGCAT | 135 |
| *16SrRNA* | ATGAAGTCGGAATCGCTAGTAATCG | CCGACTTCGGGTGTTGCAAAC | 111 |
